# Biomarkers of Chronic Inflammatory State in Uremia and Cardiovascular Disease

**DOI:** 10.1155/2012/360147

**Published:** 2012-06-04

**Authors:** Vincenzo Panichi, Alessia Scatena, Massimiliano Migliori, Valentina Marchetti, Sabrina Paoletti, Sara Beati

**Affiliations:** Nephrology and Dialysis Unit, Versilia Hospital, Via Aurelia 335, 55034 Lido di Camaiore, Italy

## Abstract

Cardiovascular disease is the leading cause of death in the general population; traditional risk factors seem inadequate to explain completely the remarkable prevalence of cardiovascular mortality and morbidity observed in the uremic population. A role for chronic inflammation has been well established in the development of atherosclerotic disease, and, on the basis of these observations, atherosclerosis might be considered an inflammatory disease. Inflammation has been implicated in the etiology of coronary artery disease in the general population, and traditional inflammatory biomarkers such as C-reactive protein (CRP) and interleukin-6 (IL-6) have been shown to predict cardiovascular events in both symptomatic and asymptomatic individuals as well as those in the uremic population. Later on, new nontraditional markers were related to the risk of cardiovascular morbidity and mortality in general and in uremic population. As a consequence of the expanding research base and availability of assays, the number of inflammatory marker tests ordered by clinicians for cardiovascular disease (CVD) risk prediction has grown rapidly and several commercial assays have become available. So, up to now we can consider that several new nontraditional markers as CD40-CD40 ligand system and pentraxin-3 seem to be significant features of cardiovascular disease in general and in ESRD population.

## 1. Introduction

Patients with end-stage kidney disease undergoing chronic hemodialysis (HD) present higher mortality rates compared with the general population. Once patients are on HD, the risk of cardiovascular death is approximately 30 times higher than that in the general population and remains 10–20 times higher after stratification for age, gender, and the presence of diabetes. About half of the deaths of patients on dialysis are attributed to cardiovascular causes including coronary heart disease, cerebrovascular disease, peripheral vascular disease, and heart failure.

End-stage renal disease (ESRD) patients suffer from a state of chronic inflammation leading to cardiovascular complications, progressive malnutrition, and death [1, 2]. Inflammation is subclinical, and chronic disorders of the cytokine system or acute-phase proteins may be observed as the sole evidence of a proinflammatory disorder.

According to this hypothesis traditional inflammatory biomarkers such as tumor necrosis factor-alfa (TNF-alfa), C-reactive protein (CRP), and interleukin-6 (IL-6) have been shown to predict cardiovascular events in both symptomatic and asymptomatic individuals as well as those in the uremic population. More recently, several new nontraditional biomarkers have been introduced in the clinical practice.

## 2. Traditional Biomarkers of Chronic Inflammation

Low-grade chronic inflammation, as indicated by levels of high-sensitivity C-reactive protein (hs-CRP), prospectively defines the risk of atherosclerotic complications, adding to the prognostic information provided by traditional risk factors. The study of Ridker et al. [3] provides convincing evidence that, in apparently healthy subjects, baseline serum levels of hs-CRP are predictive of future myocardial infarction and ischemic stroke. Subsequent meta-analysis of prospective population-based studies has compared patients in the lower tertile of hs-CRP with those in the upper tertile [4, 5]. With a good consistency between studies, a higher risk for major coronary events was observed for the upper tertile with the lowest tertile used as a reference. In general population most studies showed a dose-response relationship between the level of hs-CRP and risk of incident coronary disease. Recent papers also suggest association with incidence of sudden death [6, 7] and peripheral arterial disease [8]. Through stratification or multivariable statistical adjustment, hs-CRP retains an independent association with incident coronary events after adjusting for age, total cholesterol, HDL cholesterol, smoking, body mass index, diabetes, history of hypertension, exercise level, and family history of coronary disease [9, 10]. In terms of prediction of recurrent CVD events and death, the strongest association with prognosis has been with hs-CRP; hs-CRP consistently predicts new coronary events in patients with unstable angina and acute myocardial infarction [11–20].

As elevated serum levels of hs-CRP have been shown to be such a strong predictor of cardiovascular mortality in the general population, available data suggest that the association between inflammation and atherosclerosis is particularly strong in uremic patients [21, 22]. Zimmermann et al. [2] reported that chronic inflammation enhances cardiovascular risk and mortality; a few years later Ikizler et al. [23] in a prospective study assessed the importance of hs-CRP values as independent determination of hospitalization in chronic hemodialysis (HD) patients.

Recently, it has been shown that proinflammatory cytokines such as IL-6 may exert a direct inflammatory effect on the heart and peripheral circulation [24]. In a previous published paper, we investigated the joint predictive power of CRP and IL-6, in order to ascertain what is the prognostic information that each index carries independently of the other. To this aim, IL-6 and CRP plasma levels were measured in a cohort of 218 ESRD patients from different centres over a 4-year followup. Main outcomes were cardiovascular and total mortality. This study showed that plasma IL-6 rather than CRP better predicts outcome in ERSD patients. Various possible explanations may underline the advantage of IL-6 over CRP as an outcome predictor. One possibility is that, being located upstream in the cascade of events which lead to the synthesis of many acute-phase reactants, IL-6 is a better marker of the inflammatory burden affecting the development of cardiovascular disease. Another possibility is that levels of IL-6 vary less than those of CRP, leading to a more accurate classification of patients at risk when one single sample is taken. Finally, the toxic effects of IL-6 on the heart and peripheral vasculature might be stronger than those of CRP [24]. This study provides some important implications. First, it gives further support to the hypothesis about the role of inflammatory mediators in the genesis of cardiovascular disease in dialysis patients [25–27]. Secondly, it provides evidence suggesting the use of IL-6 in addition to, or even in place of, CRP for the identification of patients at risk.

Zhang et al. [28] reported that there was no association between CRP haplotypes and cardiovascular outcome in dialysis patients; this study argues against CRP as a cardiovascular risk factor. On the other hand, because variations within the IL-6 gene were shown to affect the risk for CVD in a multiethnic dialysis cohort [29], this suggests that IL-6 should be the target for interventional studies.

According to these data, we suggest that all traditional risk factors for death should be measured accurately in uremic patients. Clinical events should be identified prospectively, and, whenever possible, IL-6 levels should be measured repeatedly during the course of followup.

TNF-alfa, a proinflammatory cytokine (17 kDa) originally associated with killing of tumor cells, has a pivotal role in regulating both pro- and anti-inflammatory mediators. TNF-alfa has been regarded a “master regulator” of the cytokine cascade that provides a rapid form of host defense against infection but is fatal in excess. TNF-alfa is highly multifunctional with effects on lipid metabolism, coagulation, insulin resistance, and endothelial dysfunction. The major cellular origin of TNF-alfa, previously known as cachectin, is activated macrophages. It should be noted that, whereas IL-6 is strongly associated with CRP and other inflammatory biomarkers, the association between TNF-alfa and CRP is rather weak. This suggests that circulating levels may be influenced by a number of different factors and that circulating TNF-alfa levels may not reflect biologic activity at the tissue levels. Although available evidence suggests upregulated TNF-alfa system activity in ESRD patients [30], data linking elevated circulating TNF-alfa levels to CVD and mortality have not been as clear as for IL-6.

## 3. Nontraditional Biomarkers of Chronic Inflammation

It is now generally accepted that CD40-CD40 ligand interaction is a main determinant of the proatherogenic phenotype [31]. Originally identified in B and T lymphocytes as being involved in T-cell-dependent B-cell activation and differentiation, the CD40-CD40 ligand system has been implicated in the pathophysiology of several chronic inflammatory diseases including risk factor-related vascular damage [32]. CD40, a 50 kDa integral membrane protein of the tumor necrosis factor receptor family, and its cognate agonist CD40 ligand also known as CD154, a transmembrane 39-kDalton protein structurally related to tumor necrosis factor-alpha, are coexpressed by several cells of the vasculature, including endothelial cells, smooth muscle cells, and macrophages [31]. CD40 ligand also occurs in a soluble form (sCD40L) that is considered to possess a full biological activity [33]. Increased sCD40L levels have been described in obesity [34], hypercholesterolemia [35], diabetes [36, 37], and unstable angina [38]. Furthermore, it has been recently reported that circulating sCD40L has a strong independent prognostic value among apparently healthy individuals [39] and patients with acute coronary syndromes [40] and represents an independent predictor of restenosis after percutaneous transluminal angioplasty [41]. Thus, the clinical association between soluble CD40L and cardiovascular events suggests that soluble CD40L function spans the time interval from early atherogenesis to late thrombotic complications.

**Figure 1:**
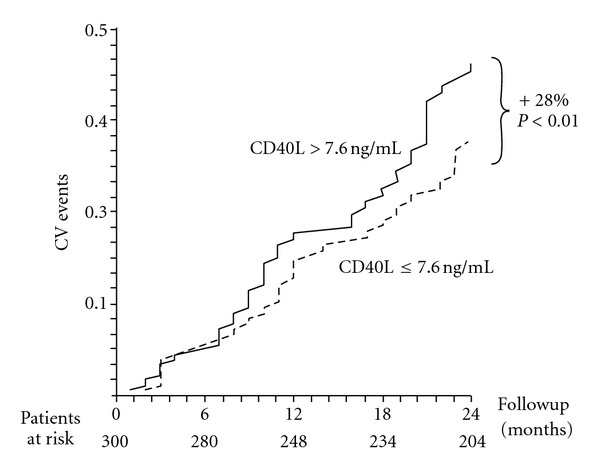
Prognostic value of CD40L in the RISCAVID population.

According to this, Hocher et al. [42] recently demonstrated during a follow-up period of 52 months that sCD40L is an independent predictor of atherothrombotic events in patients on HD. More recently we expanded on this topic demonstrating that the prognostic value of sCD40L is evident also in over 200 chronic HD patients from the RISCAVID population at 24-month followup [43] (RISCAVID, “risk cardiovascular in dialysis” is a prospective observational study performed on a large HD population in the northwestern region of Tuscany, Italy) (Figure 1).

In this paper we were able to demonstrate that this prognostic value of sCD40L is already evident at 24 months followup thus reinforcing the strong link between sCD40L and clinical outcomes in patients in HD and suggesting a possible clinical use of this new promising biomarker to better define cardiovascular prognosis in these patients. The striking prognostic impact of sCD40L on the clinical course in patients in HD raises questions about the origin of this biomarker. Platelets represent the main source of circulating sCD40L in patients with acute coronary syndrome [38] and in hypercholesterolemia [35]. Accordingly, plasma levels of sCD40L correlate closely with markers of platelet activation in these patient populations [35, 38]. Thus, increased circulating levels of sCD40L might reflect an enhanced platelet activation in HD. According to this, it has been demonstrated that circulating activated platelets (P-selectin/CD63-positive platelets) are higher in HD patients than in controls and further increase during HD sessions [44]. Potential causes of such activation include possible stimulation of platelets by proinflammatory cytokines that have been reported to be increased in patients with end-stage renal disease [45]. Furthermore, the increased lipid peroxidation that has been found in patients with chronic renal failure might also participate in activating platelets [46]. On the other hand, the lack of any correlation between circulating levels of sCD40L and CRP seems to exclude a role for this platelet-activating inflammatory biomarker [47] in the enhanced sCD40L signaling observed in our study population.

**Figure 2:**
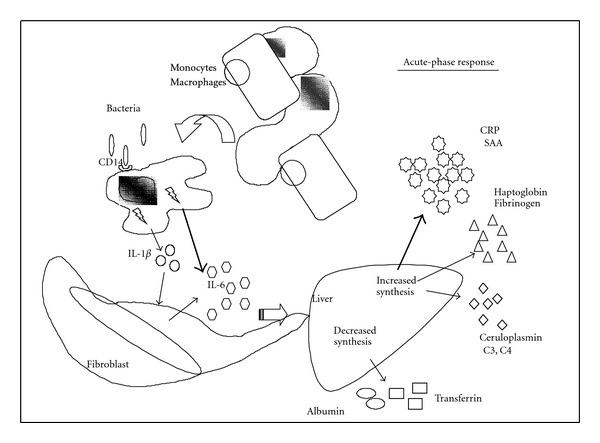
Acute phase of inflammation.

Pentraxin is a family of proteins considered to be markers of the acute-phase inflammation [48, 49] (Figure 2). Currently, the pentraxin protein family is divided into two subfamilies based on size: the classical “short” pentraxin (25 kDa) and the “long” pentraxin (40–50 kDa). Pentraxin 3 (PTX3) is a “long” pentraxin that is highly expressed in the heart, whereas C-reactive protein (CRP) is a “short” pentraxin and is produced from the liver [50]. PTX-3 expression occurs in a variety of cell types, including endothelial cells, mononuclear phagocytes, dendritic cells, smooth muscle cells, fibroblasts, adipocytes, and epithelial cells in response to inflammatory cytokines and Toll-like receptor engagement [51–53]. In several recent studies [54, 55] PTX3 appeared to be not only an early indicator of irreversible myocyte injury but also a prognostic marker in patients with acute myocardial infarction. Latini et al. [56] reported the acute-phase protein PTX3 as a predictor of 3-month mortality after adjustment for major risk factors and other acute-phase prognostic markers. In a recently published paper of Barbui et al. [57], the role of PTX3 as a prognostic biomarker was shown by an increased serum PTX3 that was closely related to death due to MI, in-hospital or to 6 months, in ACS patients, including STEMI, NSTEMI, and UAP groups. More recently, Suliman et al. [58] analyzed plasma PTX-3 concentrations in relation to comorbidities (Davies score), protein-energy wasting (PEW), and inflammation markers in 200 prevalent HD patients, aged  64 ± 14  years, who had been on HD treatment for a median period of 36 months. Survival (42 months) was analyzed in relation to PTX-3 levels (high PTX-3 tertile versus two lower tertiles). This study shows that high levels of PTX-3 were found in prevalent HD patients with CVD and PEW; furthermore, a powerful association of PTX-3 with comorbidities was founded. As PTX-3 predicts mortality independent of age and comorbidities in prevalent HD patients, further designed studies addressing the clinical implication and pathogenic mechanisms of this long pentraxin are warranted.

## 4. Conclusions

Although the successful introduction of dialysis in the 1960s has increased life expectancy in patients with ESRD, the mortality rate is still unacceptably high, due primary to a process of inflammation-associated accelerated atherosclerosis. The accelerated atherosclerotic process of ESRD may involve several interrelated processes, such as oxidative stress, endothelial dysfunction, vascular calcification, and inflammation. The explosion of new knowledge on the central role of a dysregulated cytokine and Th system activity has opened new and exciting opportunities for nephrologists to manage and prevent CVD and wasting in this diseased patient group. The use of several traditional and new biomarkers of inflammatory and cardiovascular risk is of great utility in this high-risk population.
